# The Influence of the Duration of Breastfeeding on the Infant’s Metabolic Epigenome

**DOI:** 10.3390/nu11061408

**Published:** 2019-06-22

**Authors:** Sara Pauwels, Lin Symons, Eva-Lynn Vanautgaerden, Manosij Ghosh, Radu Corneliu Duca, Bram Bekaert, Kathleen Freson, Inge Huybrechts, Sabine A. S. Langie, Gudrun Koppen, Roland Devlieger, Lode Godderis

**Affiliations:** 1 Department of Public Health and Primary Care, Environment and Health, KU Leuven—University of Leuven, 3000 Leuven, Belgium; linsymons@hotmail.com (L.S.); eva-lynn_vanautgaerden@hotmail.com (E.-L.V.); Manosij.ghosh@kuleuven.be (M.G.); radu.duca@kuleuven.be (R.C.D.); lode.godderis@kuleuven.be (L.G.); 2 VITO-Health, 2400 Mol, Belgium; sabine.langie@vito.be (S.A.S.L.); Gudrun.koppen@vito.be (G.K.); 3 Department of Imaging & Pathology, KU Leuven—University of Leuven, 3000 Leuven, Belgium; bram.bekaert@uzleuven.be; 4 Laboratory of Forensic Genetics and Molecular Archeology, Department of Forensic Medicine, University Hospitals Leuven, KU Leuven—University of Leuven, 3000 Leuven, Belgium; 5 Center for Molecular and Vascular Biology, KU Leuven—University of Leuven, 3000 Leuven, Belgium; Kathleen.freson@kuleuven.be; 6 Nutrition and Metabolism Section, International Agency for Research on Cancer, 69372 Lyon CEDEX 08, France; HuybrechtsI@iarc.fr; 7 Centre for Environmental Sciences, Hasselt University, 3590 Diepenbeek, Belgium; 8 Department of Development and Regeneration, KU Leuven—University of Leuven, 3000 Leuven, Belgium; roland.devlieger@kuleuven.be; 9 Department of Obstetrics and Gynecology, University Hospitals of Leuven, 3000 Leuven, Belgium; 10 IDEWE, External Service for Prevention and Protection at Work, 3001 Heverlee, Belgium

**Keywords:** breastfeeding, DNA methylation, *RXRA*, *LEP*, obesity

## Abstract

Nutrition in the postnatal period is associated with metabolic programming. One of the presumed underlying mechanisms involves epigenetic modifications (e.g., DNA methylation). Breastfeeding has an unknown impact on DNA methylation at a young age. Within the Maternal Nutrition and Offspring’s Epigenome (MANOE) study, we assessed the effect of breastfeeding duration on infant growth and buccal methylation in obesity-related genes (*n* = 101). A significant difference was found between infant growth and buccal *RXRA* and *LEP* methylation at 12 months of breastfeeding. For *RXRA* CpG2 methylation, a positive association was found with duration of breastfeeding (slope = 0.217; 95% confidence interval (CI) 1.03, 0.330; *p* < 0.001). For *RXRA* CpG3 and CpG, mean methylation levels were significantly lower when children were breastfed for 4–6 months compared to non-breastfed children (only CpG3), and those breastfed for 7–9 months, 10–12 months, or 1–3 months. On the other hand, higher *LEP* CpG3 methylation was observed when mothers breastfed 7–9 months (6.1%) as compared to breastfeeding for 1–3 months (4.3%; *p* = 0.007) and 10–12 months (4.6%; *p* = 0.04). In addition, we observed that infant weight was significantly lower when children were breastfed for 10–12 months. Breastfeeding duration was associated with epigenetic variations in *RXRA* and *LEP* at 12 months and with infant biometry/growth. Our results support the hypothesis that breastfeeding could induce epigenetic changes in infants.

## 1. Introduction

The concept of the “first 1000 days” assumes that the period from conception until two years of age is the most susceptible period in life, where environmental factors can induce a higher predisposition to chronic health problems. At birth, several physiological and metabolic mechanisms are not fully mature and continue to develop in the early postnatal period. Nutritional exposures during early infancy can affect normal development and may lead to permanent alterations resulting in a higher risk of childhood obesity, as well as later in life (metabolic programming) [1,2]. The World Health Organization (WHO) recommends exclusive breastfeeding for the first six months of life, with breastfeeding continuing to be an important part of the diet until the infant is at least two years old [3]. It is commonly accepted that breastfeeding has a protective effect; however, studies show both protective and null effects of breastfeeding on obesity development [4,5,6,7]. The source of the beneficial effects of human milk may be the feeding behavior associated with breastfeeding (feeding on demand gives better appetite control), slower infant weight gain (associated with a lower obesity risk), or the unique composition of breast milk. Hence, human milk has the ideal combination of hormones (leptin), nutrients (lower protein and energy levels than formula milk), and other factors (immunoglobulins) which are necessary for the health and development of babies [8].

One of the underlying mechanisms could be through the influence of breast milk on epigenetic processes like DNA methylation [8,9,10]. Only a few studies investigated the effect of breastfeeding on infant DNA methylation status [10]. To our knowledge, only one study assessed the effect of the duration of breastfeeding on the infant’s epigenome. Obermann-Borst et al. [9] found a negative association between the duration of breastfeeding and leptin (*LEP*) methylation in whole blood of 17-month-old infants. The hypothesis is that breast milk composition could change *LEP* methylation, which could contribute to programming of the neuro-endocrine system. The decrease in *LEP* methylation, which is expected to lead to increased expression of leptin and, thus, inhibition of hunger, could be one of the mechanisms via which breastfeeding contributes to protection against childhood obesity.

In this study, we investigated the effect of the duration of breastfeeding on gene-specific methylation (DNA methyltransferase 1, *DNMT1;* insulin-like growth factor 2 differentially methylated region, *IGF2* DMR; retinoid X receptor alpha, *RXRA*; leptin, *LEP)* in buccal epithelial cells of 12-month-old children. The genes were selected based on previous studies and because of their role in fat metabolism, appetite control, growth, and maintenance of DNA methylation marks (*RXRA, LEP, IGF2,* and *DNMT1,* respectively). In addition, we collected data on infant growth and investigated the association with infant DNA methylation levels and breastfeeding duration.

## 2. Materials and Methods

### 2.1. Study Subjects

**Figure 1:**
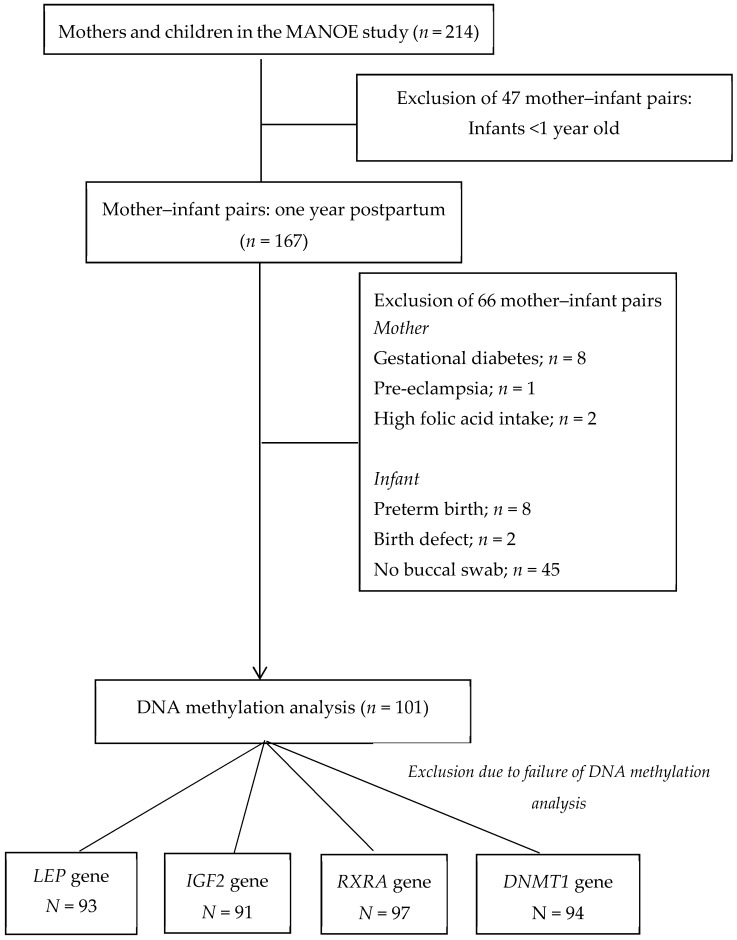
Flowchart of mothers and children recruited in the Maternal Nutrition and Offspring’s Epigenome (MANOE) study.

We studied mothers and infants recruited in the MANOE (Maternal Nutrition and Offspring’s Epigenome) study, a prospective, observational cohort study. We recruited healthy Caucasian women with a pregnancy wish or women in the first trimester of pregnancy at the Department of Obstetrics and Gynecology of the University Hospitals Leuven (Belgium). We enrolled 214 women between April 2012 and May 2018. Of the 214 enrolled women, 167 mother–infant pairs finished the study and had a one-year postpartum consultation. Sixty-six mother–infant pairs were excluded due to the following criteria: no buccal swab; the mother developed a pregnancy complication; pre-term delivery; extreme high intake of folic acid; and birth defects. This resulted in 101 mother–infant pairs for DNA methylation analysis. For some samples, DNA methylation analysis failed; thus, the statistical analysis was performed on 93 mother–infant pairs for the *LEP* gene, 91 for the *IGF2* gene, 97 for the *RXRA* gene, and 94 for the *DNMT1* gene. Figure 1 shows a flowchart of the mothers and children recruited in the MANOE study. A more detailed recruitment process was described in a previous study [11].

The MANOE study was conducted according to the guidelines laid down in the Declaration of Helsinki. All procedures involving human subjects were approved by the UZ Leuven Committee for Medical Ethics (reference number: ML7975). We received written informed consent from all subjects.

### 2.2. Maternal and Infant Measurements

A follow-up of the mother and child was planned six weeks, six months, and one year postpartum (PP). We collected information, through questionnaires and interviews, about socio-demographic factors, lifestyle habits, and physical activity. Height and pre-pregnancy weight were used to calculate the pre-pregnancy body mass index (kg/m²). Maternal measurements were described in more detail in a previous paper [11]. 

At birth, information about birth weight, length, delivery type, and type of feeding was obtained from the hospital clinical record. At the one-year PP visit, we measured infant weight and length to calculate gender specific *z*-scores for weight, BMI for age, conditional growth (from birth until one year), and weight for length using the Belgian 2004 Growth Reference Chart [12]. In addition, information about the duration of breastfeeding was obtained. The infants were divided into five categories depending on the duration of breastfeeding, which means how many months a mother breastfed with or without complementary feeding (0 = no breastfeeding; 1 = ≤1–3 months breastfeeding; 2 = 4–6 months breastfeeding; 3 = 7–9 months breastfeeding; 4 = 10–12 months breastfeeding). The infants were also divided into two breastfeeding groups according to the WHO classification (1 = <6 months breastfeeding; 2 = ≥6 months breastfeeding) [13].

### 2.3. Sample Collection and DNA Methylation Analysis

A Cytobrush plus Medscand^®^ (Medipost, Luik, Belgium) was used to brush against the inner cheeks. DNA extraction was performed using the QIAamp DNA Blood Mini Kit (Qiagen Inc., Valencia, CA, USA).

Genomic DNA was bisulfite-converted using the EZ-96 DNA Methylation-Gold™ Kit. We ordered primers from Qiagen for *DNMT1*, *RXRA*, and *LEP*. For *IGF2* DMR, we used the primer sequences described in the original paper [14].

Pyrosequencing was performed using Pyro Gold reagents on the PyroMark Q24 instrument. Pyrosequencing results were analyzed using the PyroMark analysis 2.0.7 software. We randomly selected five samples for technical variation analysis, and we added a control sample with 100% methylated DNA to each plate. Sample collection and analysis were described in more detail in a previous paper [15].

### 2.4. Statistical Analysis

Firstly, we performed an independent *t*-test and one-way ANOVA to assess whether differences in anthropometric measurements of the children (weight, weight *z*-score, weight-for-length *z*-score, conditional growth *z*-score, and BMI-for-age *z*-score) depended on the duration of breastfeeding. For the independent *t*-test, infants were divided into two categories (<6 months breastfeeding or ≥6 months breastfeeding). For the one-way ANOVA, infants were divided into five categories (0 = no breastfeeding; 1 = ≤1–3 months; 2 = 4–6 months; 3 = 7–9 months; 4 = 10–12 months). Post hoc tests (least significant difference (LSD)) were run when an overall significant difference in group means was found.

Secondly, an independent sample *t*-test was used to compare buccal DNA methylation levels (*RXRA, IGF2 DMR, LEP, DNMT1*) between children who were breastfed less or longer than six months. In addition, we assessed whether there were differences in buccal gene-specific DNA methylation depending on the duration of breastfeeding (five categories) using one-way ANOVA. Post hoc tests (LSD) were run when an overall significant difference in group means was observed.

Thirdly, we determined the effect of the duration of breastfeeding on gene-specific DNA methylation of the one-year-old children using linear mixed models. DNA methylation was used as a response variable, and duration of breastfeeding, CpG site, and the interaction between the two were used as explanatory variables. Potential confounders were chosen depending on the association with offspring DNA methylation levels and duration of breastfeeding: age of the mother, pre-pregnancy BMI, smoking (0 = no smoking during breastfeeding; 1 = smoking during breastfeeding), weight gain during pregnancy, and mode of delivery (1 = vaginal; 2 = caesarean section). Firstly, the interaction between the duration of breastfeeding and CpG site was assessed. A significant interaction test implies that the association between the duration of breastfeeding and CpG methylation is different between the individual CpGs. In the case of a significant test, individual CpG results were reported.

Finally, an independent *t*-test was performed to compare buccal DNA methylation levels between children who had a normal BMI-for-age/weight-for-length *z*-score (*z*-score between −2 and +1) and children who had a high BMI-for-age/weight-for-length *z*-score (>1 = overweight and >2 = obese).

All tests were two-sided, and a 5% significance level was assumed for all tests. Analyses were performed using SPSS software (IBM SPSS Statistics 25 for Windows).

## 3. Results

### 3.1. Description of the Study Population

Characteristics of the 101 mother–infant pairs are presented in Table 1. The mean maternal age was 30.76 years, the mean pre-pregnancy BMI was 23 kg/m^2^, and the mean gestational weight was 14.49 kg. The one-year-old infants, 58 of which were boys (57.4%), had a mean weight of 9877.23 g, a mean length of 75.2 cm, a mean weight *z*-score of 0.08, a mean BMI-for-age *z*-score of 0.39, a mean weight-for-length *z*-score of 0.37, a mean conditional growth *z*-score of −0.04, and a mean age and SD of 12.20 months.

The infants were divided into five categories depending on the duration of breastfeeding Seventeen women (16.7%) breastfed their infant for 10 or more than 10 months, while most women breastfed their infant for three or less than three months (30.7%) (Table 2).

### 3.2. Duration of Breastfeeding and Anthropometric Measurements

The one-year-old infants were divided into two breastfeeding groups. The first group consisted of 61 infants (33 boys/28 girls) who were breastfed for less than six months. The second group consisted of 40 infants (25 boys/15 girls) who were breastfed for more than six months. The characteristics of the breastfed infants are presented in Table 3. There were no significant differences between the characteristics of the two breastfeeding groups.

We found statistically significant differences in weight *z*-scores, weight-for-length *z*-scores, and BMI-for-age *z*-scores by duration of breastfeeding (five categories). The results are shown in Table 4. Firstly, we found a statistically significant difference in weight *z*-scores (*p* = 0.03) between the breastfeeding groups. A post hoc test revealed that weight *z*-scores were significantly lower when the children were breastfed for 10–12 months (−0.549 ± 0.878) compared to children who were not breastfed (0.532 ± 1.068, *p* = 0.03), and children who were breastfed for 4–6 months (0.323 ± 0.827; *p* = 0.006) and 7–9 months (0.191 ± 1.016; *p* = 0.03). Secondly, we found a statistically significant difference in weight-for-length *z*-scores and BMI-for-age *z*-scores between the breastfeeding groups (*p* = 0.005 and *p* = 0.003). A post hoc test revealed that the weight-for-length *z*-scores and BMI-for-age *z*-scores were significantly lower when the children were breastfed for 1–3 months (−0.032 ± 1.026 and −0.057 ± 1.029) or 10–12 months (−0.207 ± 0.796 and −1.8 ± 0.793) compared to children who were breastfed for 4–6 months (0.7063 ± 0.851; *p* = 0.001/*p* = 0.004, and 0.7521 ± 0.89; *p* = 0.001/*p* = 0.004) and children who were breastfed for 7–9 months (0.6306 ± 0.934; *p* = 0.02/*p* = 0.02, and 0.6675 ± 0.956; *p* = 0.01/*p* = 0.02).

### 3.3. Impact of Breastfeeding on Infant Buccal DNA Methylation

**Figure 2:**
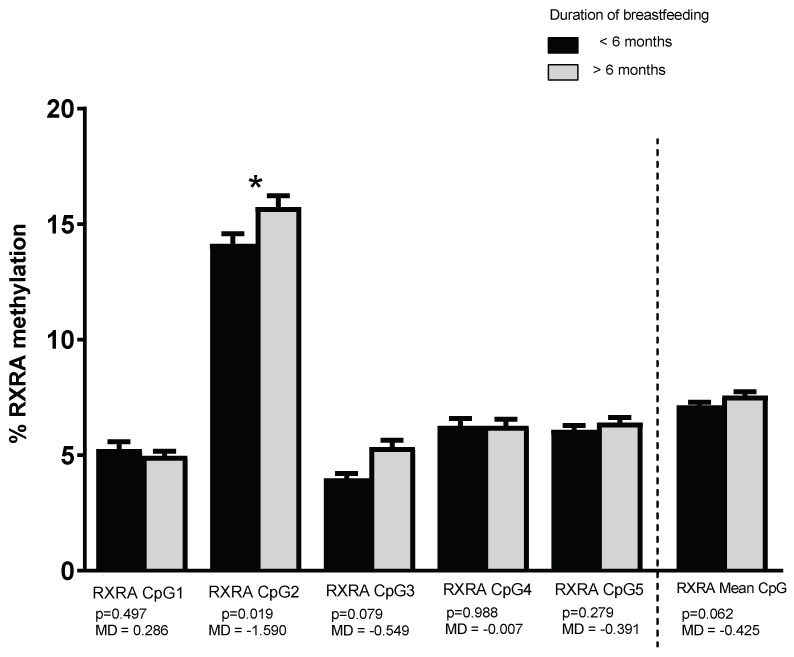
Buccal *RXRA* methylation by duration of breastfeeding. The bars represent the mean methylation values and standard errors of the mean of the 97 one-year-olds. The results are based on the duration (two categories) of maternal breastfeeding. The *p*-values and mean differences (MD) are shown. * *p* ≤ 0.05.

Firstly, we compared buccal DNA methylation levels between two groups at one year PP, namely children who were breastfed for less than six months and children who were breastfed for more than six months. For *RXRA*, we found statistically significant methylation differences between the two groups for *RXRA* CpG2. *RXRA* CpG2 methylation was significantly lower when children were breastfed for less than six months (14.14 ± 3.21%, *p* = 0.02) compared to more than six months (15.73 ± 3.06%) (Figure 2). For *LEP*, *IGF2*, and *DNMT1*, no significant differences were found between the two breastfeeding groups.

**Figure 3:**
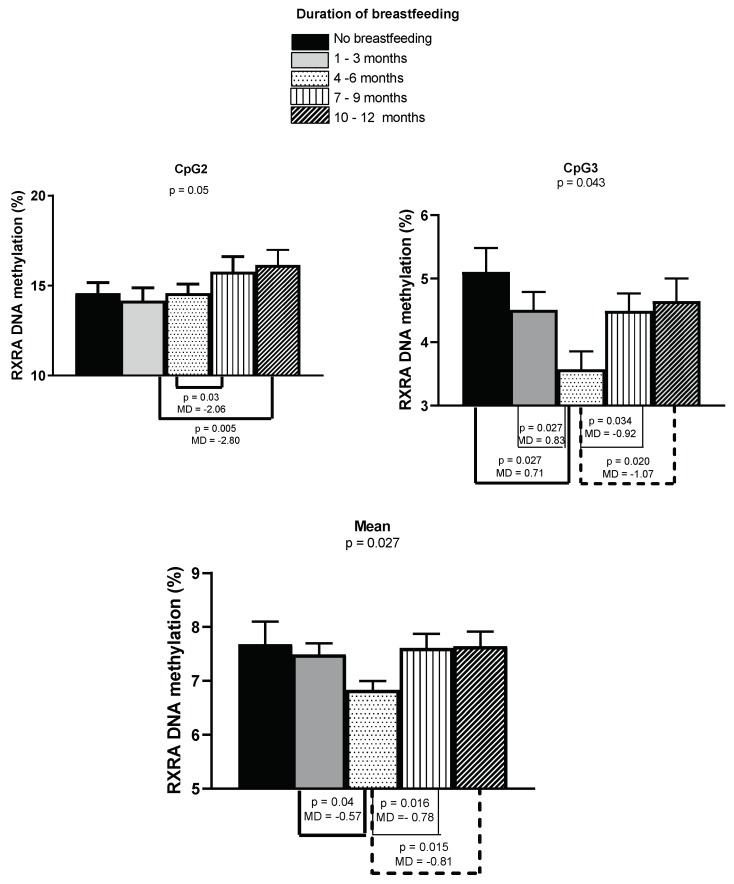
Buccal *RXRA* methylation by duration of breastfeeding. The bars represent the mean methylation values and standard errors of the mean of the 97 one-year-olds. The results are based on the duration (five categories) of maternal breastfeeding. The overall *p*-values (one-way ANOVA) and significant *p*-values with mean differences from post hoc tests are shown.

We found statistically significant differences in buccal *RXRA* CpG2, CpG3, and mean methylation by duration of breastfeeding (five categories). The results are shown in Figure 3. For *RXRA* CpG2, we found a statistically significant difference between the five groups (*p* = 0.05). A post hoc test revealed that the methylation percentage was significantly lower when the children were breastfed for 4–6 months (13.5 ± 3.6%) compared to children who were breastfed for 7–9 months (15.6 ± 2.7%; *p* = 0.03) and more than 10 months (16.3 ± 3.7%; *p* = 0.005). For *RXRA* CpG3 and mean, we found a statistically significant difference between the five groups (*p* = 0.04/*p* = 0.05). A post hoc test revealed that the methylation percentage was significantly lower when the children were breastfed for 4–6 months (3.6 ± 1.5% and 6.8 ± 0.9%) compared to children who were not breastfed (only CpG3, 5.1 ± 0.8%, *p* = 0.03), and children who were breastfed for 1–3 months (4.5 ± 1.5%; *p* = 0.03, and 7.5 ± 1.1%; *p* = 0.04), 7–9 months (4.5 ± 1.2%; *p* = 0.03, and 7.6 ± 1.1%; *p* = 0.02), and more than 10 months (4.6 ± 1.4%; *p* = 0.02, and 7.6 ± 1.1%; *p* = 0.02). 

**Figure 4:**
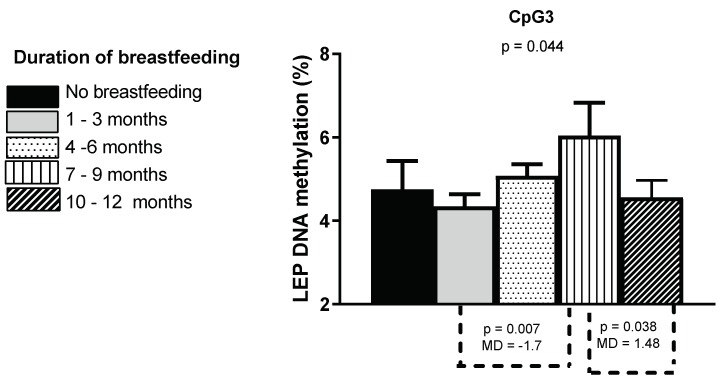
Buccal *LEP* CpG3 methylation by duration of breastfeeding. The bars represent the mean methylation values and standard errors of the mean of the 93 one-year-olds. The results are based on the duration (five categories) of maternal breastfeeding. The overall *p*-values (one-way ANOVA) and significant *p*-values with mean differences from post hoc tests are shown.

In addition, we found statistically significant differences in buccal *LEP* CpG3 methylation by duration of breastfeeding (five categories). The results are shown in Figure 4. For *LEP* CpG3, we found a statistically significant difference between the four groups (*p* = 0.04). A post hoc test revealed that the methylation percentage was significantly higher when the children were breastfed for 7–9 months (6.1 ± 3.3%) compared to children who were breastfed for 1–3 months (4.3 ± 1.5%; *p* = 0.007) and more than 10 months (4.6 ± 1.6%; *p* = 0.04).

Finally, we calculated the association of the duration of breastfeeding on infant *DNMT1*, *IGF2* DMR, *RXRA*, and *LEP* methylation levels in buccal cells. The results are presented in Table 5. The duration of breastfeeding was associated with buccal *RXRA* methylation levels. A longer duration of breastfeeding was associated with higher *RXRA* CpG2 methylation (0.217% increase in *RXRA* CpG2 methylation per extra month of breastfeeding; 95% confidence interval (CI): 0.103, 0.330; *p* < 0.001). 

### 3.4. Anthropometric Measurements and Buccal DNA Methylation

We compared buccal DNA methylation levels between two groups at one -year PP, namely children who had a normal BMI-for-age/weight-for-length *z*-score and children who had a high BMI-for-age/weight-for-length *z*-score (overweight and obese children). No significant differences in DNA methylation were found between the normal weight and overweight/obese group.

## 4. Discussion

This research supports the hypothesis that breastfeeding could induce epigenetic effects in obesity-related genes of one-year-olds. We observed that there was an association between breastfeeding duration and buccal DNA methylation in genes related to metabolism (*RXRA*, positive association) and appetite control (*LEP*, negative association).

Children who were breastfed for 4–6 months had a significantly lower methylation of *RXRA* (CpG3 and mean) compared to children who were not breastfed, and children who were breastfed for 1–3 months, 7–9 months, or 10–12 months. For *RXRA* CpG2, there was a 0.217% increase in methylation per extra month of breastfeeding. The observed higher *RXRA* methylation levels are not in line with Godfrey et al. [17], who found that methylation of the *RXRA* promoter in cord blood was positively associated with adiposity in nine-year-old children. However, the results are difficult to compare as different tissues and timings were used for DNA methylation analysis (cord blood vs. buccal epithelial cells) and we analyzed adiposity/growth at a different time point in life (nine-year-old vs. one-year-old children). Moreover, when we look at the anthropometric data of our study, we found that the breastfeeding group with the lowest *RXRA* methylation (4–6 months of breastfeeding) had the highest weight and weight/weight-for-length/BMI-for-age *z*-score at the age of one. This could mean that higher buccal *RXRA* methylation levels are associated with lower weight at one year, which is in line with a potential protective effect of breastfeeding. We speculate that changing from breastmilk to formula milk when the children are 4–6 months old could induce higher weight in the one-year-olds. Several studies already found that a higher weight gain during infancy is associated with childhood and adult obesity [18,19,20]. This is a period when most mothers in Belgium return to work, stop breastfeeding, introduce formula milk, and start with complementary feeding. Shifting from breastmilk to formula milk in this period may have a negative effect on metabolic development and DNA methylation levels due to higher protein/calorie content of formula milk, lack of self-regulation in intake, or different composition of methyl-group donors in formula milk [18].

Next, we found differences in buccal *LEP* methylation at one year of age depending on the duration of breastfeeding. We found that *LEP* methylation was lowest when children were breastfed for 10–12 months or 1–3 months compared to 7–9 months. Obermann-Borst et al. [9] also found a negative association between the duration of breastfeeding and *LEP* methylation of whole-blood samples of 17-month-old children. These results are in line with our results, namely that breastfeeding for 10–12 months is associated with lower *LEP* methylation. There are two theories that demonstrate a link between breastfeeding, metabolic programming, and epigenetic mechanisms [21]. The “early protein hypothesis” states that the higher protein and calorie content in formula milk is associated with rapid weight gain in infancy and can program children for obesity through epigenetic alterations [7,22]. The second theory is the “leptin deficiency theory”. Human milk contains many hormones and growth factors. The hormone leptin, secreted by adipocytes, has a known role in the energy balance and is present in breast milk (but absent in formula milk). Leptin concentration in breast milk decreases slowly during the first 180 days [23]. Breast-fed infants have higher serum leptin levels than formula-fed infants, and serum leptin levels in breastfed infants are positively correlated with the leptin contents of their mothers’ breast milk [23,24]. Leptin acts on the brain during a critical window in the postnatal period to establish neurological pathways to regulate food intake and energy consumption for life, which could be mediated through epigenetic alterations [9,24,25]. The leptin gene is involved in the development of obesity. Lower methylation of the *LEP* promoter leads to a higher leptin expression and, thus, a higher concentration of leptin. Higher leptin levels are associated with adiposity in young children and neonates [26,27]. In our cohort, contrary to our expectations, the one-year old children with the lowest weight, weight-for-length, and BMI-for-age *z*-scores were from the breastfeeding groups (10–12 months and ≤1–3 months) with the lowest *LEP* methylation. We expected to find higher *LEP* methylation levels because of the association with adiposity [27]. Obermann-Borst et al. [9] also found that an increased weight at birth and childhood BMI were associated with lower *LEP* methylation levels.

The MANOE study had some strengths and limitations. The first strength was the unique study design that allowed us to collect longitudinal maternal data (three time points in the postnatal period where we asked about breastfeeding) and gene-specific DNA methylation levels in buccal epithelial cells of 12-month-old children. We had detailed covariate data allowing us to adjust for potential confounding variables. The use of bisulfite pyrosequencing for gene-specific DNA methylation analysis was another advantage. DNA methylation levels at individual CpG sites could be determined, and the average methylation percentage of that region could be calculated. Single CpG site methylation in the promoter region of a gene can be involved in the regulation of transcription, especially when it lies in a relevant transcription factor binding site, and this methylation could be associated with diseases. CpG methylation within the same CpG island in promoter regions was shown to be highly correlated, and these methylation patterns were shown to differ from methylation patterns elsewhere, indicating that they have a specific biological role [28]. A first limitation was that we did not measure methylation in the adipose tissue (target tissue) but, rather, in buccal epithelial cells. We do not know to what extent the methylation changes found in buccal epithelial cells reflect the changes in adipose tissue. Buccal DNA mainly stems from exfoliated epithelial cells, which have a more homogeneous cell population compared to blood samples. Non-invasive methods, like buccal swabs, are often used in epidemiological studies involving young children [29]. It was shown that buccal samples are more informative than blood samples in DNA methylation studies with non-blood-based diseases/phenotypes (for example, obesity) as the outcome [30,31]. One last limitation of the MANOE study was that we only had information on breastfeeding duration, defined as the age at which the infant ceased to receive any breast milk. Since an inverse relationship between breastfeeding duration and infant overweight/obesity was observed, future studies need to go into more detail by obtaining information about the period of exclusive breastfeeding, if breast milk was given from the breast or bottle, the introduction of complementary feeding, etc., since these factors might have on impact on the infant [32].

## 5. Conclusions

This study suggests that breastfeeding can influence infant growth and offspring buccal DNA methylation levels in metabolism- and appetite regulation-related genes. Longer breastfeeding could have an effect on childhood obesity development, which might be explained by an upregulation of *RXRA* and a downregulation of *LEP* in one-year old children.

## Figures and Tables

**Table 1:** Maternal and infant characteristics (*n* = 101). BMI—body mass index.

Characteristics	Mean (SD)	Range
Mother		
Maternal age (y)	30.76 (3.65)	24–41
Pre-pregnancy BMI (kg/m^2^)	23.00 (3.25)	17.93–32.95
Gestational weight gain (kg)	14.49 (4.05)	7–28.9
Infant (1-year-old)		
Weight (g)	9877.23 (1089.37)	7980–13 200
Length (cm)	75.20 (2.53)	71–82.5
Age (months)	12.20 (0.35)	11.56–13.23
Weight *z*-score *	0.08 (0.97)	−2.24–2.60
BMI-for-age *z*-score *	0.39 (1.02)	−1.71–3.14
Weight-for-length *z*-score *	0.37 (1)	−1.75–2.98
Conditional growth *z*-score *	−0.04 (0.89)	−2.68–2.45
	**%**	***N***
Pre-pregnancy BMI (kg/m^2^) **		
Underweight (<18.5)	3.0	3
Normal weight (18.5–24.9)	71.3	72
Overweight (25.0–29.9)	22.8	23
Obese (>30.0)	3.0	3
Maternal smoking (yes)		
During pregnancy/breastfeeding	4	4
Type of Delivery		
Vaginal	75.2	76
Caesarean section	24.8	25
Gender		
Boy	57.4	58
Girl	42.6	43

* Gender-specific *z*-scores were generated using the Belgian 2004 Growth Reference Chart [12]. ** BMI ranges according to the World Health Organization (WHO) [16].

**Table 2:** Distribution of breastfeeding duration (*n* = 101).

Duration of Breastfeeding	*N*	%
No breastfeeding	5	5.0
≤1–3 months	31	30.7
4–6 months	29	28.7
7–9 months	19	18.8
10–12 months	17	16.8

**Table 3:** Characteristics of the infants based on duration of breastfeeding (*n* = 101). PP—postpartum.

Infant Characteristics 1 Year PP	Duration of Breastfeeding		*p*-Value
	<6 Months (*n* = 61) Mean (SD)	≥6 Months (*n* = 40) Mean (SD)	
Age (months)	12.20 (0.33)	12.19 (0.39)	0.79
Length (cm)	75.35 (2.37)	74.96 (2.77)	0.45
Weight (g)	9827.87 (996.84)	9952.5 (1226.6)	0.58
Weight *z*-score *	0.07 (0.91)	0.1 (1.06)	0.88
BMI *z*-score *	0.30 (1.02)	0.52 (1.01)	0.28
Weight-for-length *z*-score *	0.29 (1)	0.49 (1)	0.35
Conditional growth *z*-score *	−0.06 (0.75)	−0.02 (1.1)	0.84

* Gender-specific *z*-scores were generated using the Belgian 2004 Growth Reference Chart [12].

**Table 4:** Anthropometric measurements of the infants based on the duration of breastfeeding (*n* = 101).

	Weight (*z*-Score)	Weight for Length (*z*-Score)	BMI for Age (*z*-Score)
**Duration of Breastfeeding**			
No breastfeeding	0.532	0.610	0.612
≤1–3 months	−0.146	**−0.032**	**−0.057**
4–6 months	0.323	0.706	0.752
7–9 months	0.191	0.631	0.668
10–12 months	**−0.549**	**−0.207**	**−0.180**
	Overall *p*-value = 0.03	Overall *p*-value = 0.005	Overall *p*-value = 0.003

The numbers in bold are the significant results.

**Table 5:** Association between breastfeeding (months) and gene-specific buccal DNA methylation in one-year-old children. CI—confidence interval.

Gene CpG site		*RXRA*					*LEP*	*IGF2*	*DNMT*
		CpG1 ^†^	CpG2 ^†^	CpG ^†^	CpG4 ^†^	CpG5 ^†^	All CpG Sites *	All CpG Sites *	All CpG Sites *
Duration of breastfeeding (months)	Β (95% CI) *p*-value	−0.012 (−0.125, 0.101) 0.83	0.217 (0.103, 0.330) <0.001	0.058 (−0.056, 0.173) 0.32	−0.005 (−0.118, 0.108) 0.93	0.03 (−0.083, 0.144) 0.60	0.060 (−0.073, 0.193) 0.37	0.229 (−0.050, 0.509) 0.12	−0.041 (−0.085, 0.003) 0.06

The β-estimate is an absolute change in percentage of methylation; slope >0 (<0) means positive (negative) association. * When there was no evidence for a differential association between the duration of breastfeeding and DNA methylation at the different CpG locations, the main effect of the duration of breastfeeding over all CpG locations was reported. ^†^ When there was a significant interaction test, the association between the duration of breastfeeding and DNA methylation was different between CpG locations. In this case, the results were reported per CpG location.
